# High HPV Infection Prevalence in Men from Infertile Couples and Lack of Relationship between Seminal HPV Infection and Sperm Quality

**DOI:** 10.1155/2014/956901

**Published:** 2014-04-06

**Authors:** Barbara Golob, Mario Poljak, Ivan Verdenik, Mojca Kolbezen Simoniti, Eda Vrtačnik Bokal, Branko Zorn

**Affiliations:** ^1^Reproductive Unit, Department of Obstetrics and Gynaecology, University Medical Centre Ljubljana, Šlajmerjeva 3, 1000 Ljubljana, Slovenia; ^2^Institute of Microbiology and Immunology, Faculty of Medicine, University of Ljubljana, Zaloška 4, 1000 Ljubljana, Slovenia

## Abstract

Human papillomaviruses (HPV) are the most frequently sexually transmitted viruses and etiological agents of several human cancers. Controversial results of the role of HPV in infertile population on sperm parameters have been published. The aim of this study was to estimate the type-specific prevalence of HPV DNA infection of the external genitalia and semen in 340 Slovenian men from infertile couples and to establish the relationship between seminal HPV DNA infection and abnormal sperm parameters. Self-taken swabs of the entire penile surface and semen samples were collected, and HPV detection and genotyping were performed. HPV DNA was detected in 37.12% of external genitalia and in 13.61% of semen samples with high HPV type concordance of both sampling sites. The most prevalent HPV types in the male external genitalia were HPV-CP6108 and HPV-84. The most prevalent HPV types in semen were HPV-53 and HPV-CP6108. The prevalence of HPV infection between normozoospermic men and men with abnormal sperm parameters did not differ significantly. Sperm quality did not differ significantly between men with seminal HPV infection and uninfected men. In conclusion, the men from infertile couples are equally susceptible to HPV infection regardless of their fertile potential; seminal HPV infection does not impair sperm quality.

## 1. Introduction

Human papillomaviruses (HPV) are the most common sexually transmitted viruses. More than three quarters of the sexually active human population will acquire an HPV infection during their lifetime, although most of the infections are self-limited [1]. Persistent HPV infection is a necessary cause of several human cancers, mainly in the anogenital area [2]. By February 2014, more than 179 HPV types have been officially recognized, and about 40 different HPV types from the clinically most important HPV genus (alpha) affect the anogenital region of both genders [3].

HPV can be detected in every part of the male reproductive tract [4–6]. Since the early reports of seminal HPV infection in the middle eighties, the role of HPV infection in infertility has been debated [7]. World Health Organization (WHO) defines infertility as an inability of a sexually active, noncontraceptive couple to achieve spontaneous pregnancy in one year [8]. Approximately 15–20% of couples in reproductive period encounter infertility problems. Many factors affect the fertility potential and they are equally distributed between genders: in 1/3 of infertile couples, a male infertility associated factor is found, in 1/3 a female associated factor is found, and in 1/3 both female and male infertility associated factors are found present [9]. Maldescended testis, varicocele, and inflammation are among the most common diagnoses of male infertility and represent one of the three most frequent causes of male infertility [10]. In spite of highly improved diagnostic procedures, about 10% of couples remain diagnosed with idiopathic infertility that does not provide a cause for their defect [8]. A connection between asthenozoospermia and HPV DNA sperm infection has been observed* in vivo* and* in vitro* [11–14]. Paradoxically, after unsuccessful sperm wash, when HPV DNA was still present in a sample, spermatozoa expressed higher motility [15]. However, the connection between a lower total sperm count and seminal HPV DNA infection has also been reported [16], whereas in another study the high-risk HPV (HR-HPV) sperm infection has been found to correlate to borderline lower pH of ejaculate [17].

HPV DNA possesses an ability of binding to the surface of viable spermatozoa [18–21]. Furthermore, viable spermatozoa have the potential of uptaking the exogenous HPV DNA fragments* in vitro* [22]. Lai et al. have shown the expression of oncogenes E6 and E7 of HPV-16 and HPV-18 separately in spermatozoa fraction and sperm plasma [23]. Foresta et al. have reported that transfected sperm is able to penetrate hamster oocytes [19].* In vitro* studies have shown some detrimental effects of HPV DNA on sperm cells and early embryo development [24–26].

On the other hand, the absence of any significant correlation between seminal HPV DNA infection and abnormal sperm parameters has been reported [21, 27, 28].

The prevalence of HPV DNA seminal infection in patients attending fertility clinics ranges between 7.8% and 28.6% [21, 28]. There is a lack of published data on the prevalence of HPV infection in external genitalia in men from infertile couples.

In the Slovenian population, HPV has been studied for over 20 years [29], mostly in women. The overall prevalence of HR-HPV infection in women (mean age 36.6 years) in the recent national HPV Prevalence Study was 12.9%, whereas an 8.2% prevalence of HR-HPV types was assessed in 195 Slovene women undergoing the IVF program [30, 31]. However, there are no data on the prevalence of HPV infection in male sub/infertile population.

Recently, HPV has been proposed as an agent possibly impairing sperm quality and function. Due to inconsistency in reporting possible adverse effects of HPV infection on semen, we designed a study enrolling a representative sample population of male patients from the infertility clinic to screen their genital and seminal samples for HPV DNA. The aim of this study was to establish the type-specific prevalence of HPV DNA infection of external genitalia and semen with 37 different alpha HPV types in a group of men from Slovenian infertile couples. Additionally, relationships between the presence of HPV DNA in semen and abnormal sperm parameters were analysed.

## 2. Materials and Methods

### 2.1. Study Design and Population

This was a cross-sectional study conducted in an outpatient infertility clinic between October 2010 and October 2013. The study protocol had been approved by the National Medical Ethics Committee (Consent Number 53/09/10). Men were recruited prospectively at the same visit when they provided semen for sperm analysis. Men were eligible for participation if they (1) were male partners from couples attending the clinic for inability to conceive within at least one year of unprotected regular sexual intercourse, (2) underwent sperm parameters analysis, and (3) provided semen by masturbation. Additionally, a clinical examination was done. Azoospermic men were excluded from the statistical analysis concerning the difference in sperm parameters between HPV infected and uninfected men.

### 2.2. Specimen Collection

After a written consent was obtained, the participants were carefully instructed to provide a semen sample and self-taken swab of the entire penile surface. They were provided with a sterile container for semen collection, a flocked nylon swab (MicroRheologics, Brescia, Italy) prewetted with saline for self-swabbing, and 1 mL of specimen transport medium (STM) (QIAgen, Gaitheburg, USA) for the swab storage. The participants were carefully instructed to wash their hands before masturbation and to swab the entire penile surface (avoiding possible sperm traces) immediately after masturbation. Self-swabbing using the prewetted swab took at least one minute to provide adequate number of exfoliated cells.

After the liquefaction of the collected semen samples, 100 *μ*L of semen samples and the entire STM with the inserted swab were frozen and stored at −20°C until PCR analyses and typing were undertaken. At the same time classical sperm parameters were analysed.

### 2.3. Sperm Analyses

Semen samples were provided by masturbation after 2–5 days of sexual abstinence. After an hour, semen was assessed according to the World Health Organization (WHO) 2010 guidelines by one of the two well-trained technicians [32]. Briefly, after initial macroscopic examination, a wet preparation was obtained for assessing microscopic appearance, sperm motility, and the dilution required for assessing sperm number. A standard volume of 10 *μ*L of semen was placed onto a clean glass slide and covered with a coverslip 24 mm × 24 mm. When the sample was spread and the contents were no longer drifting, the progressive and nonprogressive motility was assessed with brightfield optics at ×400 magnification. Many fields were viewed and the motility was displayed as an average proportion of instantly estimated sperm motility in those many fields. Afterwards, semen was diluted with fixative (1 : 5 or 1 : 20) for assessing sperm concentration. An improved Neubauer haemocytometer of 100-*μ*m-deep haemocytometer chambers was used. The method of feathering (the semen drop was spread along the back edge of the angled slide and pulled forwards over the slide to form the smear) was used for preparation of semen smears for assessing sperm morphology. The air-dried smears were fixed with ethanol and stained using the Papanicolaou procedure. The slides were examined with brightfield optics at ×1000 magnification with oil immersion and 100 spermatozoa were assessed for the percentage of normal and abnormal forms. For sperm morphology evaluation, the strict criteria were used. The men were considered normozoospermic when sperm concentration was ≥15 × 10^6^ spermatozoa/mL, progressive motility ≥32%, and the percentage of spermatozoa with normal morphology ≥4% (representing the 5th centile, lower reference limits proposed by WHO 2010) [33]. The men whose sperm concentration, motility, and the percentage of spermatozoa with normal morphology were below the lower reference limits were considered having abnormal sperm parameters.

### 2.4. Isolation of DNA

Total DNA was isolated from STM and semen samples using EZ1 Virus Mini Kit (QIAgen, Valencia, CA, USA) on BioRobot EZ1 workstation (QIAgen) according to the manufacturer's instructions.

DNA was eluted in the Elution buffer (QIAgen) and stored at 4°C.

### 2.5. PCR and HPV DNA Typing

HPV DNA amplification, detection, and typing were performed using the commercially available Linear Array HPV Genotyping Test (Roche Molecular Diagnostics, Pleasanton, CA) capable of detecting 37 different alpha-HPV types, following the manufacturer's instructions. Prior to PCR amplification using PCR GeneAmp 9700 (Life Technologies Corporation, Carlsbad, USA) concentrations of DNA in all isolates from semen samples were estimated by spectrophotometric analysis at 260 nm using a NanoDrop 2000c spectrophotometer (Thermo Fisher Scientific, Wilmington, DE). 250 ng of DNA per final volume of 50 *μ*L PCR reaction was used. The isolates from swabbed samples were loaded in a maximum volume (25 *μ*L) per 50 *μ*L PCR reaction.

Hybridization and PCR detection were performed using automated processor ProfiBlot T48 (Tecan, Salzburg, AT). Types 16, 18, 31, 33, 35, 39, 45, 51, 52, 56, 58, and 59 were considered as high-risk (HR) types [34]. Samples were considered valid if they were HPV-positive by genotyping or *β*-globin positive, regardless of HPV results. When HPV infection was subdivided on the basis of the risk of type of HPV, the men were assigned into a HR group, if they were infected with at least one HR type. Linear Array HPV Genotyping test uses multiple type probes (one cross-reactive oligonucleotide probe that hybridizes with HPV-33, HPV-35, HPV-52, and HPV-58) to detect DNA from HPV-52 infection; thus, we were unable to determine the HPV-52 status in the presence of HPV-33, HPV-35, or HPV-58 infection. In statistical analysis, such cases were considered as HPV-52 negative.

### 2.6. Statistical Analysis

Statistical analysis was performed using IBM SPSS Statistics 20 software (IBM Corporation, USA). General descriptive data of men with normozoospermia and abnormal sperm parameters and the values of sperm parameters were presented as arithmetic mean ± standard deviation. Chi-Square statistic was used for the evaluation of differences in the HPV prevalence between normozoospermic men and men with abnormal sperm parameters. Mann-Whitney test was performed to compare sperm parameters between men with seminal HPV infection and uninfected men. Kruskal-Wallis test was performed to compare sperm parameters between HPV uninfected men, men with LR-HPV type of seminal infection, and men whose sperm was infected with at least one of HR-HPV. When the difference was significant, linear regression was performed, and age, abstinence, number of leukocytes, orchiopexy, testicular trauma, varicocele, and testicular volume were used as covariables.

## 3. Results

### 3.1. Population and Samples Adequacy

Overall, 340 men were enrolled, their mean age being 32.91 ± 5.15 years. Mean values of age, duration of infertility, number of smoked cigarettes per day, testicular volume, and sperm parameters for a group of men with normozoospermia and a group of men with abnormal sperm parameters are summarized in Table 1. Forty-one samples of swabbed penile surface and 24 semen samples were not provided properly or the amplification of internal control (*β*-globin) failed.

### 3.2. Prevalence of HPV DNA Infection of Penile Surface

**Figure 1:**
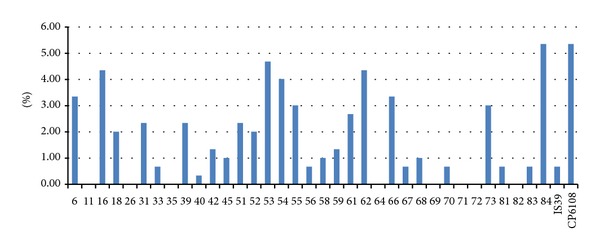
Type-specific prevalence of HPV infection of penile surface in men from infertile couples (*n* = 299).

The prevalence of HPV DNA infection of the penile surface was 37.12% (111/299). The most prevalent HPV types on the penile surface were HPV-CP6108 and HPV-84 with type-specific prevalence 5.35% each, followed by HPV-53, HPV-16, HPV-62, and HPV-54, each detected in 4.01%–4.68% of samples (Figure 1, Table 3). Prophylactic vaccine types HPV-16, HPV-18, HPV-6, and HPV-11 were detected in 4.35%, 2.01%, 3.34%, and 0% of the penile surface samples, respectively. 45.45% of all HPV infected men presented HR-HPV in samples of the swabbed penile surface; 45.95% of all detected HPV DNA infections were multitype infections.

### 3.3. Prevalence of HPV DNA Infection in Semen

**Figure 2:**
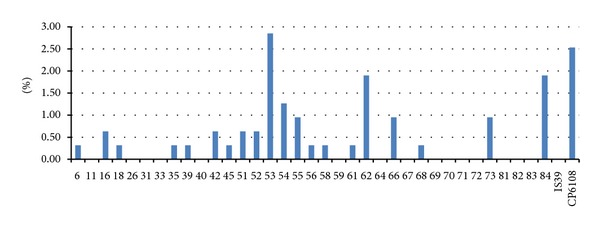
Type-specific prevalence of HPV infection of semen in men from infertile couples (*n* = 316).

The prevalence of HPV DNA infection in semen was 13.61% (43/316). The most prevalent types in semen were HPV-53 and HPV-CP6108 with the type-specific prevalence of 2.85% and 2.53%, respectively, followed by HPV-84, HPV-54, and HPV-62, each detected in 1.27%–1.90% (Figure 2, Table 4). Prophylactic HPV-16, HPV-18, HPV-6, and HPV-11 vaccine types were detected in 0.63%, 0.32%, 0.32%, and 0% of the semen samples, respectively. In three of the four seminal infections, low-risk HPV (LR-HPV) types were present. Multitype infection was observed in 27.91% of all infections.

### 3.4. HPV DNA Infection in Normozoospermic Men versus Men with Abnormal Sperm Parameters

Men from the total cohort were subdivided according to sperm parameters in a normozoospermic group and a group of men with abnormal sperm parameters; 246 (72.4%) men were considered normozoospermic, and 94 (27.6%) men had abnormal sperm parameters. Semen analysis of 4.1% (14/340) of men showed azoospermia, and 23.5% (80/340) of men had combined oligoasthenoteratozoospermia (OAT). Oligozoospermia and/or asthenozoospermia and/or teratozoospermia, asthenozoospermia and/or oligozoospermia and/or teratozoospermia, and teratozoospermia and/or oligozoospermia and/or asthenozoospermia were observed in 71, 52, and 42 men, respectively. Isolated oligozoospermia, asthenozoospermia, and teratozoospermia were observed in 30 (8.8%), 13 (3.8%), and 6 (1.8%) men, respectively. No significant difference in seminal HPV DNA infections was observed between the normozoospermic group and the group of men with abnormal sperm parameters (Table 2).

### 3.5. Concordance of HPV Type Infection among Genital and Seminal (Paired) Samples of the Same Men

Concordance of at least one of the detected HPV types was found in 30.51% (36/118) of paired samples. In 58.47% (69/118) of paired samples, only one of the two checked samples contained HPV DNA. We were unable to determine the concordance in 11.02% (13/118) of paired samples, since one of the paired samples was HPV-positive and the other was inhibited. The negative concordance for all types was 50.59%. The total HPV type-specific concordance was 60.59%.

### 3.6. The Effect of Seminal HPV DNA Infection on Sperm Parameters

No statistically significant difference was found in sperm quality between men with seminal HPV infection and uninfected men. The difference in sperm quality between HPV uninfected men and men with HR- or LR-HPV seminal infection was not statistically significant.

## 4. Discussion

The relationship between HPV infection in men and abnormal sperm quality is controversial. As long as routine sperm washing fails to eliminate HPV DNA from the ejaculate, it is of crucial importance to determine whether HPV DNA sperm infection affects the quality of sperm parameters or not [16, 35]. The aim of this study was to establish the type-specific prevalence of HPV DNA infection of external genitalia and semen with at least one of the 37 most important alpha HPV types in a group of men from Slovenian infertile couples. Additionally, relationships between the presence of HPV DNA in semen and abnormal sperm parameters were studied.

### 4.1. Genital Surface HPV Prevalence

The sample size of the study population was calculated prior to recruitment, the men were recruited systematically, and the selection bias was minimal; therefore we may consider our study sample representative. We found the overall prevalence of HPV infection in men from infertile couples to be 40.28%, regardless of the sampling site and sperm quality. Since the penile surface (shaft, preputium, frenulum, coronal sulcus, and glans) is frequently infected with HPV, our first aim was to determine the genital HPV prevalence in men from infertile couples [36]. The obtained prevalence of HPV infection of the penile surface was 37.03%. Since men underwent semen analysis after two to five days of sexual abstinence, we assume that the detected infection was not a contamination from their female partners. Self-swabbing for a minute and immediately after masturbation is supposed to increase the prevalence. Self-swabbing has been reported to be as efficient as sampling by a physician [37]. The detection of an active infection, without a possibility of acquiring new or reactivated infection at a delayed visit, was assured by collecting samples from the penile surface and semen at the same visit [38]. It is difficult to make direct comparisons of the genital HPV prevalence between the published studies. To the best of our knowledge, this is the first study reporting the genital HPV prevalence in men from infertile couples. HPV prevalence among men has been reported to range from 1.3% to 72.9% [1]. In a recent study from Florida dealing with heterosexual couples aged 18–70 years, the genital HPV prevalence in males for any type was 55.7% [39]. In the HPV in Men Study that enrolled men having sex with men, men having sex with women and men, and men having sex with women (MSW), the genital HPV prevalence among MSW was 42% with the peak in the group aged 25–34 years [40]. Another recent study reported a 41.7% HPV DNA prevalence in a cohort of 1033 males (median age 34 years) who were screened for sexually transmitted diseases, investigation of suspected HPV-related lesions, or because of HPV-positive partners [41]. With respect to the latter, genital HPV prevalence in men from infertile couples seems to be particularly similar to the genital HPV prevalence among the general age-related population of MSW, even if the population of infertile couples could be speculated to differ in sexual behaviour and promiscuity from general heterosexual couples. The mean duration of infertility in couples of participants in our study was almost two years, supposing that the duration of their stable sexual relationships was even longer. Taking into account the clearance time of HPV infection, which is generally shorter than two years in men, we expected much lower HPV prevalence [42].

The most prevalent HPV types in the penile surface samples of men from infertile couples were HPV-84 and HPV-CP6108. The latter is a LR-HPV type and one of the most frequently detected HPV types in men [36], but rarely present in women [41]. HPV-84 is a HPV type without clearly established clinical significance that has a relatively high prevalence [6, 36, 41]. Half of the HPV infections of the penile surface were caused by LR-HPV types. Interestingly, in contrast to HPV-6, HPV-11 was not detected in any sample tested.

### 4.2. Seminal HPV Prevalence

The prevalence of seminal HPV infection differs greatly between different groups of men, from 10.2% in infertile men to 53.8% in men with genital warts [14]. In our study, seminal HPV infection was detected in 13.38% of men from infertile couples. The prevalence is in concordance with that established in similar studies in which men from infertile couples were enrolled [14, 21]. The most frequent types detected were HPV-53 and HPV-CP6108, followed by HPV-84, HPV-54, and HPV-62, respectively. Comparative evaluation of the type-specific HPV DNA prevalence of the penile surface and concordant semen samples showed that the most frequent HPV types on the penile surface were also among the most frequent types and, moreover, in almost the same order as in semen samples. High concordance of at least one of the detected HPV types in both samples might be explained by the observation provided by a study performed in France in 2002, where patients with a positive HPV semen sample and penile or urethral lesions had the same HPV type detected in the two specimens [43]. In our study, almost one-third of men had the same HPV type on the penile surface and in the semen. It is widely known that the preferred target of HPV is the squamous epithelium [44]. Since squamous epithelium is present in the male reproductive tract only in the distal 2 cm of the urethra, this could be a possible source of seminal infection/contamination with the same type as detected in a penile surface sample [45]. If HPV might affect the sperm quality, the virus should not only contaminate but also cohabit with spermatozoa under certain conditions [18]. Multitype infections were less likely to be detected in seminal samples than in penile surface samples. Three of the four HPV types detected in semen were LR-HPV types. The latter might suggest a selective mechanism of the seminal HPV infection.

Slovenia has integrated the HPV vaccination into its national immunization program in 2009 and currently provides routine vaccination free of charge only to the primary target population (11- to 12-year-old girls) [46]. The prevalence of the HPV types covered by quadrivalent vaccine is expected to decline among primary target population in the next decades; however, current HPV national vaccination program does not affect infertile couples. Almost half of the HPV-infected men in our study had at least one of the HR-HPVs on the swabbed penile surface. Accordingly, the great majority of female partners of (at least) the HPV-infected men are supposed to be nonnaïve for specific HPV types, if not also currently infected [39]. Although not covered by government, it will be worthy to offer HPV vaccination to all females from infertile couples, at least to partners of HPV-negative males.

### 4.3. HPV Infection in Men with Different Sperm Quality

Male reproductive capacity was found to be deficient in not less than 50% of infertile couples evaluated [8, 9]. By the new “WHO laboratory manual for the examination and processing of human semen,” normozoospermia is defined as sperm concentration, motility, and morphologically normal spermatozoa that are equal to or above the lower reference limits. When the 1999 WHO reference values were used, approximately 40% of men from infertile couples were reported to have an abnormal sperm parameter [33]. The introduction of the new WHO reference values in 2010 resulted in 15% of infertile men being reclassified as fertile, if the status is based on semen analysis alone [47]. In a recent study, the proportion of sperm abnormalities (pH, sperm volume, concentration, total sperm count, and motility) was compared between WHO 1999 and WHO 2010 classification: abnormal sperm parameters according to WHO 2010 reference values were found only in 25.4% of 571 men from couples undergoing fertility investigations [48]. However, in our study, 27.6% of men from infertile couples could have been considered infertile according to the WHO 2010 reference values of sperm analysis. Interestingly, converting the WHO 2010 to WHO 1999 reference values would bring 168 (49.4%) men from our study having abnormal sperm parameters. For men from infertile couples, it seems important to know whether the seminal HPV infection affects their sperm potential or not, regardless of the more or less arbitrary reference values. The number of men with isolated sperm defects was too low to allow for a credible statistical analysis, but the prevalence of HPV infection of the penile surface and semen in the total group of men that had abnormal sperm parameters was not significantly different from that in normozoospermic men (not even, if WHO 1999 reference values would have been used).

In statistical analysis, when HPV infection prevalence was compared between normozoospermic men and the total group of men with abnormal sperm parameters, men with azoospermia were excluded. However, it is interesting to note that four azoospermic men were diagnosed with the HPV DNA infection of the penile surface, one of them having additional HPV infection in the ejaculate. All of them had histologically confirmed nonobstructive azoospermia (Sertoli cell-only syndrome with early maturation arrest). The presence of HPV DNA in testicular biopsies from nonobstructive azoospermic men had previously been reported, but there is lack of data on HPV DNA presence in ejaculated sperm [5]. In the sample of ejaculate of only azoospermic men with HPV infection of the penile surface and semen, HPV-CP6108 was detected. It is possible that the source of the detected seminal type was contamination from the genital surface, where multitype infection with HPV-6, HPV-39, HPV-51, and HPV-CP6108 was detected. However, the presence of infected nonsperm cells in the ejaculate could not be ruled out. Therefore, it seems that all men are equally susceptible to HPV infection, regardless of their fertile potential and that seminal HPV infection does not impair sperm quality.

### 4.4. The Difference in Sperm Parameters between HPV Infected and Uninfected Men

In the present study, we were unable to detect any significant difference in a variety of sperm parameters previously reported as impairing the sperm quality, between men having seminal HPV infection and uninfected men. The results of our study imply that HPV infection does not seem to affect sperm quality in terms of causing clinically significant alterations of sperm parameters. Similar results have been demonstrated in a study involving couples undergoing IVF treatment and were screened for the HPV-16 DNA infection [27]. Moreover, semen samples of men seeking fertility evaluation were screened for any type of HPV infection [28]. The prevalence of seminal infection in the latter study was higher than in the present study; however, no significant difference in sperm quality was found between infected and infected men. In a recent study, 308 semen samples of male partners of couples undergoing IVF treatment were screened for the HR-HPV DNA infection [21]. The HPV DNA infection did not significantly differ between infected and uninfected men. In our study, we have not observed any clinically significant alteration of sperm parameters, which is contrary to some authors who have reported an association between seminal HPV infection and decreased sperm motility or reduced pH of seminal plasma [11, 13, 16, 17]. The studies reporting on the differences in sperm quality between infected and uninfected men are inconsistent, not providing useful information on which sperm parameter would be most indicative of HPV infection. Furthermore, there have been some opposite effects of seminal HPV infection on sperm parameters reported; some groups have demonstrated decreased sperm motility in HPV infected men, whereas the* in vitro* studies have found that normal spermatozoa have higher motility after incubation with specific HR-HPV DNA [15, 49]. A possible explanation for these discrepancies may be a small and selected study size, narrow range of tested HPV types, or coinfection with other urogenital infections.

## 5. Conclusion

To the best of our knowledge this is the first study where the genotype-specific prevalence of HPV DNA infection of external genitalia of men from infertile couples has been estimated. HPV DNA has been detected in more than one-third of external genitalia samples and one-eighth of semen samples in men from infertile couples with a mean duration of infertility of almost two years. The HPV type-specific concordance of genital and seminal infection exceeds 60%. No significant differences in the prevalence of HPV DNA infection between groups of normozoospermic men and men with abnormal sperm parameters have been found. Similarly, we have not found any significant difference in sperm quality between the men with seminal HPV infection and uninfected men. The results of our study suggest that the presence of HPV does not impair sperm quality.

## Figures and Tables

**Table 1:** Mean values of characteristics of men from infertile couples, *n* = 340.

	Normozoospermia, *n* = 246	Abnormal sperm parameters, *n* = 94
Age (years)	33.2 ± 4.8	32.2 ± 5.9
Duration of infertility (years)	1.9 ± 1.0	1.9 ± 1.5
Smoking (number of cigarettes per day)	4.6 ± 6.7	4.6 ± 8.4
Testicular volume-left (mL)	20.6 ± 4.1	16.0 ± 6.0
Testicular volume-right (mL)	21.2 ± 4.2	16.1 ± 6.5
Abstinence (days)	4.4 ± 3.8	3.7 ± 1.5
Sperm volume (mL)	3.3 ± 1.3	3.6 ± 1.7
pH	7.7 ± 0.2	7.8 ± 0.2
Motility a + b (%)	53.0 ± 7.0	26.6 ± 18.4
Concentration (spermatozoa/mL)	74.4 ± 40.2	13.3 ± 17.7
Total sperm count	241.4 ± 161.5	42.9 ± 64.7
Spermatozoa with normal morphology (%)	24.6 ± 12.8	7.8 ± 11.8
Leukocytes (per mL)	0.68 ± 1.2	0.75 ± 1.0

**Table 2:** Prevalence of seminal HPV DNA infection in men from infertile couples according to their sperm parameters.

	Normozoospermia	Abnormal sperm parameters				*p* value
		O	A	T	OAT	
HPV DNA infection of semen	24/160 (15.0%)	2/24 (8.3%)	1/11 (9.1%)	1/6 (16.7%)	3/30 (10.0%)	NS

O: oligozoospermia; A: asthenozoospermia; T: teratozoospermia; OAT: oligoasthenoteratozoospermia; NS: not significant (*p* > 0.05).

**Table 3:** Type-specific prevalence of HPV in samples from penile surface (*n* = 297).

HPV type	Type-specific prevalence	Number
CP6108	5.35%	16
84	5.35%	16
53	4.68%	14
62	4.35%	13
16	4.35%	13
54	4.01%	12
66	3.34%	10
6	3.34%	10
73	3.01%	9
55	3.01%	9
61	2.68%	8
51	2.34%	7
39	2.34%	7
31	2.34%	7
52	2.01%	6
18	2.01%	6
59	1.34%	4
42	1.34%	4
68	1.00%	3
58	1.00%	3
45	1.00%	3
IS39	0.67%	2
83	0.67%	2
81	0.67%	2
67	0.67%	2
56	0.67%	2
33	0.67%	2
70	0.67%	2
40	0.33%	1
72	0.00%	0
71	0.00%	0
69	0.00%	0
64	0.00%	0
82	0.00%	0
35	0.00%	0
26	0.00%	0
11	0.00%	0

**Table 4:** Type-specific prevalence of HPV in semen samples (*n* = 316).

HPV type	Type-specific prevalence	Number
53	2.85%	9
CP6108	2.53%	8
62	1.90%	6
84	1.27%	6
54	1.27%	4
55	0.96%	3
66	0.96%	3
73	0.96%	3
16	0.63%	2
42	0.63%	2
51	0.63%	2
52	0.63%	2
6	0.32%	1
18	0.32%	1
35	0.32%	1
39	0.32%	1
45	0.32%	1
56	0.32%	1
58	0.32%	1
61	0.32%	1
68	0.32%	1
11	0.00%	0
26	0.00%	0
31	0.00%	0
33	0.00%	0
40	0.00%	0
59	0.00%	0
64	0.00%	0
67	0.00%	0
69	0.00%	0
70	0.00%	0
71	0.00%	0
72	0.00%	0
81	0.00%	0
82	0.00%	0
83	0.00%	0
IS39	0.00%	0
